# Genetically Encoded XTEN‐based Hydrogels with Tunable Viscoelasticity and Biodegradability for Injectable Cell Therapies

**DOI:** 10.1002/advs.202301708

**Published:** 2024-03-13

**Authors:** Jennifer I. Bennett, Mary O'Kelly Boit, Nicole E. Gregorio, Fan Zhang, Ryan D. Kibler, Jack W. Hoye, Olivia Prado, Peter B. Rapp, Charles E. Murry, Kelly R. Stevens, Cole A. DeForest

**Affiliations:** ^1^ Department of Chemical Engineering University of Washington Seattle WA 98105 USA; ^2^ Department of Bioengineering University of Washington Seattle WA 98105 USA; ^3^ Department of Biochemistry University of Washington Seattle WA 98105 USA; ^4^ Institute for Protein Design University of Washington Seattle WA 98105 USA; ^5^ Flagship Labs 83, Inc. 135 Morrissey Blvd. Boston MA 02125 USA; ^6^ Institute of Stem Cell & Regenerative Medicine University of Washington Seattle WA 98109 USA; ^7^ Department of Laboratory Medicine & Pathology University of Washington Seattle WA 98195 USA; ^8^ Department of Medicine/Cardiology University of Washington Seattle WA 98109 USA; ^9^ Department of Chemistry University of Washington Seattle WA 98105 USA; ^10^ Molecular Engineering & Sciences Institute University of Washington Seattle WA 98105 USA

**Keywords:** biomaterial, biopolymer, coiled‐coil, hydrogels, injectable, protein, XTEN

## Abstract

While direct cell transplantation holds great promise in treating many debilitating diseases, poor cell survival and engraftment following injection have limited effective clinical translation. Though injectable biomaterials offer protection against membrane‐damaging extensional flow and supply a supportive 3D environment in vivo that ultimately improves cell retention and therapeutic costs, most are created from synthetic or naturally harvested polymers that are immunogenic and/or chemically ill‐defined. This work presents a shear‐thinning and self‐healing telechelic recombinant protein‐based hydrogel designed around XTEN – a well‐expressible, non‐immunogenic, and intrinsically disordered polypeptide previously evolved as a genetically encoded alternative to PEGylation to “eXTENd” the in vivo half‐life of fused protein therapeutics. By flanking XTEN with self‐associating coil domains derived from cartilage oligomeric matrix protein, single‐component physically crosslinked hydrogels exhibiting rapid shear thinning and self‐healing through homopentameric coiled‐coil bundling are formed. Individual and combined point mutations that variably stabilize coil association enables a straightforward method to genetically program material viscoelasticity and biodegradability. Finally, these materials protect and sustain viability of encapsulated human fibroblasts, hepatocytes, embryonic kidney (HEK), and embryonic stem‐cell‐derived cardiomyocytes (hESC‐CMs) through culture, injection, and transcutaneous implantation in mice. These injectable XTEN‐based hydrogels show promise for both in vitro cell culture and in vivo cell transplantation applications.

## Graphical abstract

Shear‐thinning and self‐healing telechelic recombinant protein‐based hydrogels based on XTEN are introduced. Point mutations to self‐associating coil domains yield materials with programmable viscoelasticity and biodegradability. These materials protect and sustain viability of human fibroblasts, hepatocytes, HEK, and embryonic stem‐cell‐derived cardiomyocytes cultured within these genetically encodable biopolymer networks following in vivo injection. Such injectable hydrogels hold promise for therapeutic cell transplantation.

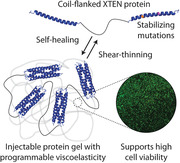

## 1 Introduction

Direct injection provides a simple and straightforward route to localize therapeutic cells precisely to diseased bodily tissues in a minimally invasive manner.^[^ ^1^, ^2^, ^3^ ^]^ This method has been extensively explored in the quest to treat many debilitating diseases including myocardial infarction, osteoarthritis, and Parkinson's.^[^ ^4^, ^5^, ^6^ ^]^ Although these therapeutic strategies exhibit great promise, engraftment and long‐term survival of the injected cells is typically quite low (<10%), dramatically limiting overall efficacy and imposing substantial barriers in cost and efficiency toward clinical translation. Such poor viability has been appropriately attributed to many factors, including cell membrane‐damaging shear forces accompanying syringe‐ and catheter‐based injection, a lack of a supportive 3D matrix and its pro‐survival signals from cell adhesion, and host inflammatory and immune responses. Methods for robust cell transplantation that address these problem areas remain in great need.

Capable of being delivered through a catheter, insulating against membrane‐damaging extensional flow, and supplying a supportive 3D environment in vivo, injectable biomaterials represent an attractive tool toward improving cell retention and subsequent tissue function following transplantation.^[^ ^7^ ^]^ Such systems are most commonly derived from synthetic polymers [e.g., poly(ethylene glycol), poly(2‐hydroxyethyl methacrylate), poly(N‐isopropylacrylamide)].^[^ ^8^, ^9^, ^10^, ^11^, ^12^, ^13^, ^14^, ^15^ ^]^ While these synthetic biomaterials afford precise physicochemical tunability, concerns persist over their potential immunogenicity, toxicity, and lack of biodegradability. Alternatively popular injectable materials have been developed from tissue‐harvested biomolecules (e.g., collagen, gelatin, alginate, Matrigel, fibrin, decellularized extracellular matrix).^[^ ^16^, ^17^, ^18^, ^19^, ^20^ ^]^ Though generally more supportive of native cell functions, these natural protein‐based platforms are limited by a lack of tunability, high polydispersity, and substantial batch‐to‐batch variability.

Biomaterials derived instead from recombinant proteins afford exciting opportunities for biomedical applications as they exhibit precise user‐defined sequence specificity, synthetic scalability through large‐scale fermentation, and intrinsic biodegradability.^[^ ^21^, ^22^ ^]^ Through inclusion of structural domains that support interprotein physical association, such recombinant systems can be designed to undergo shear‐thinning and rapid self‐healing that affords their direct injectability. In this regard, Tirrell and others have pioneered the use of intrinsically disordered protein polymers end‐functionalized with self‐associating coil domains. To date, these telechelic protein polymers have been largely based on elastin‐like polypeptides (ELPs) featuring specified repeats of the VPGXG pentapeptide (where X is any amino acid except proline, with its identity influencing its temperature transition).^[^ ^23^ ^]^ Though ELPs have found success in many applications,^[^ ^24^, ^25^, ^26^, ^27^, ^28^, ^29^, ^30^, ^31^ ^]^ including those in vivo,^[^ ^32^, ^33^, ^34^, ^35^, ^36^ ^]^ there have also been reports that ELPs can be immunogenic.^[^ ^37^, ^38^ ^]^ In addition, ELPs undergo a temperature‐dependent inverse phase transition that must be considered in an application‐specific context. While such telechelic protein‐based biomaterials are well described in the literature, surprisingly few studies have investigated their ability to support direct cell injection.^[^ ^39^ ^]^

Toward creation of an injectable protein‐based biomaterial that supports live‐cell transplantation with limited immunogenicity, we sought to create a recombinant telechelic protein platform based on XTEN – a highly expressed, water‐soluble, and chemically stable intrinsically disordered polypeptide previously evolved to “eXTENd” the in vivo half‐life of fused peptides/protein therapeutics.^[^ ^40^ ^]^ Comprised of 36‐residue pseudorepeats of only six amino acids (A, E, G, P, S, and T), XTEN lacks hydrophobic residues typically associated with histocompatibility complex class II‐driven immune responses, amino acids that typically bind cell membranes, and cysteines that can undergo disulfide crosslinking. Though XTEN has been demonstrated as a biological alternative to PEGylation and as a fusion partner to aid in soluble protein expression,^[^ ^40^, ^41^, ^42^, ^43^ ^]^ it has not been previously utilized as a material crosslinker or for bulk biomaterial creation.

In this work, we introduce a shear‐thinning and self‐healing XTEN‐based hydrogel biomaterial that shields encapsulated cells from the damaging effects of extensional flow during injection. We flank XTEN with helical end blocks (denoted P, derived from the N‐terminal fragment of rat cartilage oligomeric matrix protein) that spontaneously associate into homopentameric coiled‐coil bundles to yield macroscopic gels. Through individual and combined point mutations that alternatively stabilize physical interactions between coil domains, we demonstrate the ability to genetically program material viscoelasticity. Finally, we establish the protective nature of these gels during cell injection and their ability to sustain high viability of encapsulated human fibroblasts, hepatocytes, embryonic kidney (HEK), and embryonic stem‐cell‐derived cardiomyocytes (hESC‐CMs), both in culture and in mice. Moving forward, we anticipate that these artificial, genetically encodable biopolymer materials will find great utility for in vitro culture and in vivo therapeutic cell transplantation.

## 2 Results and Discussion

### 2.1 Protein Expression, Purification, and Characterization

**Figure 1:**
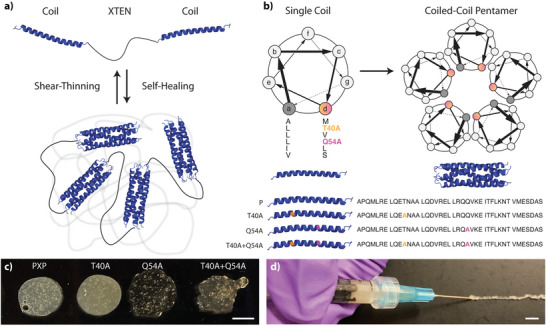
XTEN‐based coiled‐coil telechelic protein hydrogels. a) XTEN flanked with physically associating coil domains exhibit shear‐thinning upon application of stress and self‐healing in its absence. b) Coiled‐coils form homopentameric bundles whose stability can be influenced through point mutations in the coil's primary sequence. c) Macroscopic gels are formed from each mutant construct. d) Gels reform rapidly following injection through a high‐gauge syringe needle, here shown for PXP. Scale bars = 500 µm.

Toward the creation of injectable biomaterials based on artificial, genetically encoded triblock ABA‐type protein copolymers, we cloned bacterial expression vectors with gene sequences encoding for XTEN flanked by self‐associating P domains, the RGD peptide to promote cell adhesion, and a 6xHis tag for affinity purification (denoted PXP). The coiled P domains are derived from the N‐terminal fragment of cartilage oligomeric matrix protein, with near‐complete sequence similarity in human, rat, and mouse, but contain two cysteine‐to‐serine mutations to prevent covalent crosslinking through disulfide bond formation.^[^ ^44^, ^45^, ^46^ ^]^ These P domains non‐covalently associate into homopentameric coiled‐coil bundles that, at high concentration, yield physically crosslinked hydrogels (**Figure** **1**a,b). To modulate the strength of these gel‐forming physical interactions, we mutated two polar residues within the parent P coil to the smaller, more hydrophobic alanine (i.e., T40A, Q54A) expected to stabilize coil‐coil interactions,^[^ ^47^, ^48^, ^49^ ^]^ both individually and together, yielding expression plasmids for proteins denoted T40A, Q54A, and T40A+Q54A (Table S1, Supporting Information). Following transformation and colony selection, we recombinantly expressed PXP, T40A, Q54A, and T40A+Q54A in *E. coli* and purified each species via immobilized metal affinity chromatography. Highly pure samples with the expected molecular masses were obtained for all proteins, as indicated by sodium dodecyl sulfate‐polyacrylamide gel electrophoresis (SDS‐PAGE) analysis and through liquid chromatography‐mass spectrometry (Figures S1–S5, Supporting Information). High yields of purified protein (>100 mg per L of culture) were obtained following endotoxin removal for each of the four species, consistent with expectation that an XTEN‐based material would be highly expressed. To confirm the stabilizing effects of the alanine mutations to the P domains, we measured the protein secondary structure content between 25 and 95 °C by circular dichroism spectroscopy. We found the double mutant to be slightly more thermostable than PXP or the single mutants (PXP < T40A < Q54A < T40A+Q54A), with PXP partially melted at 25 and 37 °C, T40A partly melted at 37 °C, and Q54A and T40A+Q54A retaining secondary structure up to 50 °C. Additionally, we observed a maximum of ≈15% helical content, consistent with the expected contributions of two helical P domains with the much longer and disordered XTEN and purification tags (Figure S6, Supporting Information).

### 2.2 Injectability and Recovery of Gels

**Figure 2:**
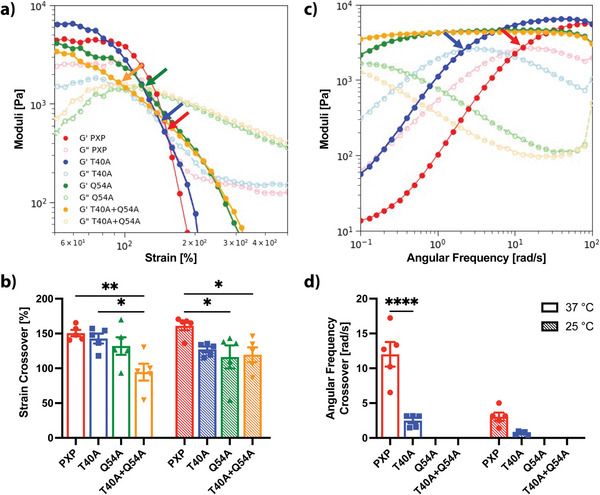
Rheometric analysis of coiled‐coil XTEN‐based hydrogels. G′: storage modulus represented by dark‐colored closed circles, G″: loss modulus represented by light‐colored open circles. PXP in red, T40A in blue, Q54A in green, and T40A+Q54A in orange. a) Representative strain sweeps for all gel types at 37 °C and 30 rad s^−1^ from 0 – 500% strain. Shear‐thinning or injectable behavior is observed for all gel types by strain crossover points (G″ > G′) indicated by colored arrows. b) Strain crossover values reported from strain sweeps showing a statistically significant decrease (*p* < 0.05) in strain crossover with the introduction of both mutations (T40A+Q54A) at 37 °C (open bars) and 25 °C (hatched bars). c) Representative frequency sweeps for all gel types at 37 °C and 5% strain from 0.1 – 100 rad s^−1^. Frequency crossover points indicated by colored arrows. A lengthened linear viscoelastic range (LVER) is observed after the introduction of point mutations as demonstrated by the length of plateau storage modulus (G′) in frequency sweeps. d) Frequency crossover reported from frequency sweeps indicating improved stability with mutations due to increased LVER (no crossover for Q54A and T40A+Q54A). Bars represent the mean of N = 5 replicates, error bars indicate SEM. A multiple comparisons two‐way ANOVA table was implemented for mutant comparison (* *p* < 0.05, ** *p* < 0.01, **** *p* < 0.0001).

Purified PXP proteins yielded stable hydrogels when reconstituted with PBS at 10% (w/v) and were macroscopically injectable through a 26‐gauge needle (26G, inner diameter = 260 µm) at room (25 °C) and physiological (37 °C) temperatures (Figure 1c,d, Figure S7, Supporting Information). To understand the viscoelastic properties of these materials, we performed in situ rheological analysis at both 25 and 37 °C (**Figure** **2**). Strain sweeps identified a strain crossover for each gel type, indicating the injectable nature of PXP and mutants due to the disruption of physical coiled‐coil‐stabilizing bonds under high strain, resulting in an elastic material at low strain (G′ > G″) and a viscous material at high strain (G′ < G″) (Figure 2a,b). The strain crossover at 37 °C occurred at 151 ± 5% strain for PXP, 143 ± 7% strain for T40A, 132 ± 13% strain for Q54A, and 95 ± 12% strain for T40A+Q54A, allowing for successful injection through a 26G needle (**Table** **1**). Intriguingly, though each point mutation substitutes a smaller hydrophobic alanine for a polar residue in a manner previously reported to yield improved P‐domain association,^[^ ^47^, ^48^, ^50^ ^]^ we observed a statistically significant decrease (*p* < 0.01) in strain crossover with the introduction of both P domain mutations (T40A+Q54A); results matched trends in mutant coil stability (Figure S6, Supporting Information).

**Table 1:** Summary of rheological properties for each gel at 25 and 37 °C. Storage modulus calculated from time sweeps (5% strain, 30 rad s^−1^). Strain crossover (G″ > G′) calculated from strain sweeps (30 rad s^−1^, 0 – 500% strain). Frequency crossover calculated from frequency sweeps (5% strain, 0.1 – 100 rad s^−1^, G′ > G″). No frequency crossover was observed for Q54A or T40A+Q54A. Recovery time crossover (G′ > G″ after high strain period) calculated from cyclic strain sweeps (30 rad s^−1^, 5% low strain, 500% high strain). Error reported as SEM, N = 5.

Parameter	Temp [°C]	PXP	T40A	Q54A	T40A+ Q54A
Storage Modulus [kPa]	25	6.1 ± 0.4	5.7 ± 0.7	3.8 ± 0.6	3.8 ± 0.3
	37	4.5 ± 0.5	6.6 ± 1.3	4.0 ± 0.9	4.5 ± 0.4
Strain Crossover [%]	25	161 ± 6	127 ± 4	116 ± 17	119 ± 11
	37	151 ± 5	143 ± 7	132 ± 13	95 ± 12
Frequency Crossover [rad s^−1^]	25	3.0 ± 0.6	0.7 ± 0.1	–	–
	37	12.0 ± 1.8	2.5 ± 0.4	–	–
Recovery Time Crossover [s]	25	4.79 ± 0.06	4.51 ± 0.05	4.45 ± 0.09	4.39 ± 0.06
	37	7.91 ± 2.23	4.58 ± 0.12	4.39 ± 0.04	4.38 ± 0.05

To further investigate the viscoelastic properties of these recombinant protein‐based materials, we performed frequency sweep rheological analysis (Figure 2c,d). Results indicated that with increasing frequencies (10^−1^ – 10 rad s^−1^), elastic properties were favored over viscous properties (G′ > G″) as higher frequencies yield less time for hydrogel components to flow. Frequency sweeps identified the angular frequency crossover, which defines the lower limit of the linear viscoelastic range (LVER) or range in which the gel‐like state of the material is favored. The LVER was extended with mutated P domains, with the crossover for T40A (2.5 ± 0.4 rad s^−1^) appearing lower than for the unmodified PXP (12.0 ± 1.8 rad s^−1^) at 37 °C (Figure 2d, Table 1). No crossover was found for Q54A or T40A+Q54A within the investigated frequency range, indicating a wider elastic‐dominated regime for these materials. We note that increased elasticity for the constructs with the stabilized coil domains is correlated with a simultaneous decrease in strain crossover, although all gels remained shear thinning. This suggests that in these ABA copolymer systems, it is difficult to increase material elasticity without a corresponding decrease in material ductility.

**Figure 3:**
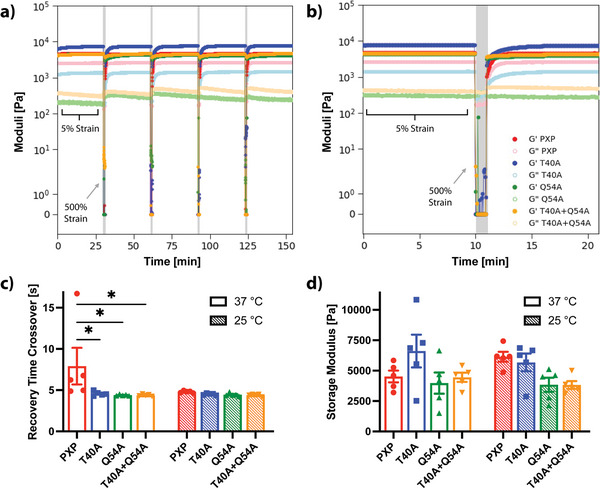
Self‐healing properties of coiled‐coil protein‐based hydrogels. G′: storage modulus represented by dark‐colored closed circles, G″: loss modulus represented by light‐colored open circles. PXP in red, T40A in blue, Q54A in green, and T40A+Q54A in orange. a) Representative cyclic strain sweep test at 37 °C and 30 rad s^−1^ with 5% low strain value and 500% high strain value (highlighted in gray). Full recovery is achieved for each gel type after each of 4 periods of high strain. b) Zoom of a representative high strain period from the cyclic strain sweep. Visually slower recovery of PXP is observed when compared to the mutants. c) Average recovery time crossover, when G′ > G″ after the high strain period, is improved for mutants by 3 s at 37 °C. d) Average storage modulus for each gel type (10% w/v) and temperature conditions with no statistical difference between mutants. Bars represent the mean of N = 5 replicates, error bars indicate SEM. A multiple comparisons two‐way ANOVA table was implemented for mutant comparison (* *p* < 0.05).

To assess their potential self‐healing behavior and recovery time, cyclic strain sweeps were performed for each gel type at 25 and 37 °C. We employed a high strain setting of 500% that was well above the crossover strain for all mutants, and a low strain setting of 5% that was well below crossover strain for all mutants. The operating frequency (30 rad s^−1^) was chosen to be within the LVER for all mutants at both temperatures. Under low strain, each material behaved as a solid with statistically indistinguishable storage moduli (G′ ≈5 kPa) across tested temperatures (**Figure** **3**, Table 1). Under high strain, the loss moduli overtook the storage moduli in all samples, indicating a near‐ instantaneous disassembly of the preformed network and a gel‐to‐sol transition. Upon return to low strain conditions, the elastic solid‐like behavior returned (G′ > G″), and a rapid recovery (typically seconds) to the initial storage modulus with minimal hysteresis was observed following 4 cycles of high strain. At 37 °C, the recovery time crossover was significantly reduced (*p* < 0.05) by ≈3 s for all mutants as compared to the original PXP, attributed to the highly favorable interactions of the stabilized mutated P domains. Collectively, these results indicate that the gel materials are capable of full recovery following high‐shear events including injection, a desirable property toward enabling local retention of therapeutic cargo following injection in living tissue.

### 2.3 Tunable Erosion Properties

**Figure 4:**
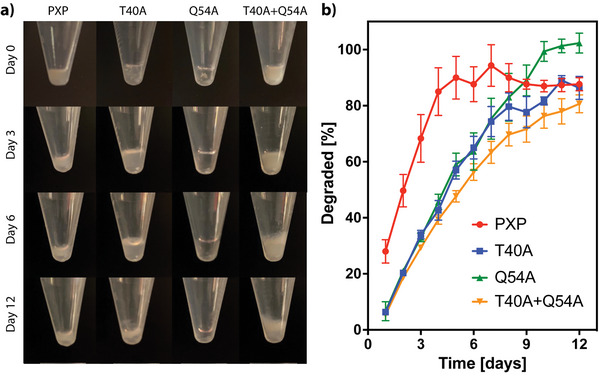
Physical erosion of XTEN‐based coiled‐coil gels in PBS. a) Photographs of each gel type following 0, 3, 6, and 12 days in PBS at 37 °C. The slowest degradation was observed in the T40A+Q54A mutant. Some of the original gel remains visually intact after 12 days for all formulations. b) BCA analysis of % gel degraded into PBS solution throughout 12 days of maintenance in PBS at 37 °C, with the PXP gels reaching plateau values significantly sooner than the mutant variants. Slower degradation is observed with the introduction of point mutations, with the slowest degradation being T40A+Q54A. Error bars reported as SEM, N = 3.

Since the designed hydrogels are non‐covalently assembled, we sought to assess their susceptibility to physical erosion and the dependence of erosion rate on the coil mutations. Gels were cast in the bottom of 15 mL Falcon conical centrifuge tubes and incubated in PBS at 37 °C for 2 weeks. The gels were monitored visually and their supernatant collected daily to assess changes in protein content as an indicator of gel degradation state over the course of 2 weeks (**Figure** **4**). All mutants (i.e., T40A, Q54A, and T40A+Q54A) yielded a slower erosion rate when compared to PXP, as seen in Figure 4 and through comparing degradation half‐lives. PXP showed a degradation half‐life of 2.1 ± 0.3 days, with mutant half‐lives as follows: T40A – 4.5 ± 0.2 days, Q54A – 4.4 ± 0.3 days, T40A+Q54A – 5.3 ± 0.2 days, where error is reported as SEM with N = 3. This confirms that the mutations stabilized physical association between P domains and allowing for tunable degradation profiles. As expected, the slowest erosion rate was observed for the material with the most stable coiled‐coil interactions – the double mutant (T40A+Q54A). Similar trends are observed when erosion studies are completed in cell culture medium (Figure S8, Supporting Information). The ability to tune the erosion rate of these artificial protein networks is an important feature in designing injectable cell/therapeutic carriers.

### 2.4 Coiled‐Coil XTEN Gels Protect Living Cells during Injection and Support Extended 3D Culture

**Figure 5:**
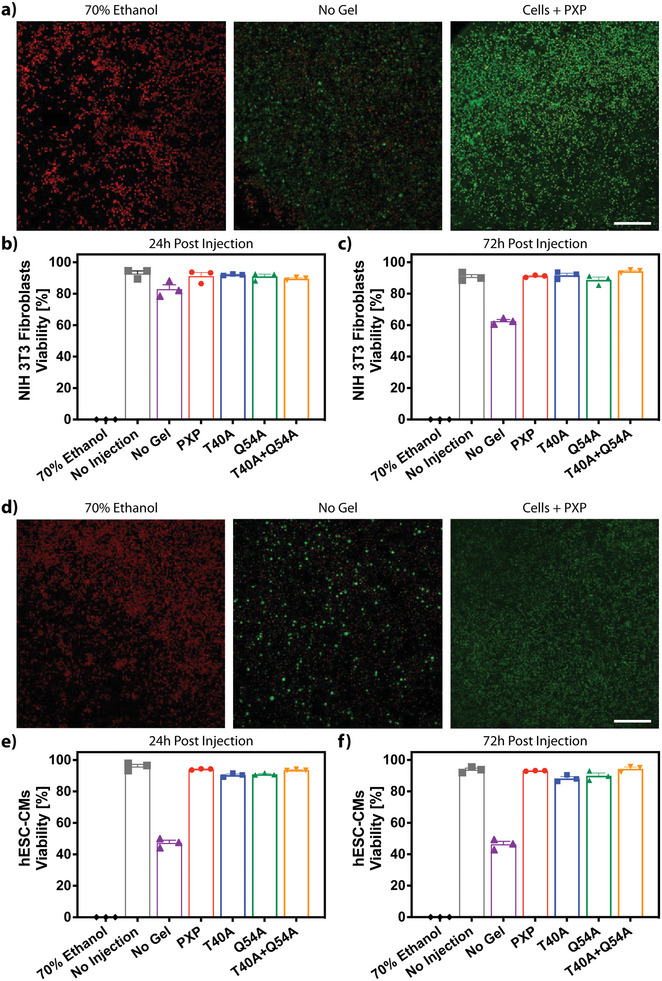
Cell viability is maintained following injection in XTEN‐based coiled‐coil gels. a) Live/dead analysis of fibroblasts 24 h following injection in 70% ethanol, media, or PXP imaged using confocal microscopy following calcein/ethidium homodimer staining. These conditions are compared to a no injection control, to demonstrate viability prior to both gel encapsulation and injection. Live cells are shown in green, dead in red. b,c) Fibroblast viability quantified through NucleoCounter analysis 24 h‐ and 72‐h post injection in the mutant gel constructs. d) Confocal live/dead analysis of hESC‐CMs 1 day following injection in 70% ethanol, media, or PXP. e,f) NucleoCounter analysis of hESC‐CMs viability 24‐ and 72‐ h following injection in all mutant gels. Error bars reported as SEM, N = 3. Scale bar = 200 µm.

To assess the suitability of our gel materials in supporting cell encapsulation, injection protection, and sustained culture, we first quantified the viability of fibroblasts (NIH 3T3) at 24‐ and 72‐h following encapsulation/injection – in each gel type (i.e., PXP, T40A, Q54A, T40A+Q54A) or as an unsupported cell suspension – via both live/dead staining and NucleoCounter analyses (**Figure** **5**a–c). Ethanol‐treated samples were included as an all‐dead sample control, and untreated cells (neither encapsulated nor injected) were included to demonstrate starting viability. Consistent with reports that cells experience membrane‐damaging forces throughout injection, a statistically significant reduction (*p* < 0.05 at 24 h, *p* < 0.0001 at 72 h) in fibroblast viability was observed for fibroblasts following in‐solution injection (26G syringe needle) compared with those that were not injected. However, we observed no significant reduction in cell viability at either timepoint when injected in an XTEN‐based gel compared with those not injected, highlighting the protective nature of the shear‐thinning recombinant protein material. Further, each mutant performed similarly well in supporting fibroblast viability and culture. These results indicate that coiled‐coil XTEN gels can be used as a delivery vehicle for injectable cell‐based therapies.

To further test the material platform's ability to protect and culture more sensitive and medically relevant cell types, we turned our attention toward utilizing the coiled‐coil XTEN gels for human embryonic stem‐cell‐derived cardiomyocyte (hESC‐CM) delivery (Figure 5d–f). Results mirrored those for cultured fibroblasts, in that hESC‐CMs exhibited no significant loss in viability at 24‐ and 72‐h following injection when encapsulated in the XTEN‐based hydrogels (i.e., PXP, T40A, Q54A, T40A+Q54A). Notably, higher losses of viability were observed when hESC‐CMs were injected as an unsupported cell suspension as compared with the 3T3s, reflecting differences in shear sensitivity between cell types. Collectively, these studies demonstrate that the coiled‐coil XTEN gels are cytocompatible and support injection of therapeutically relevant cells with high viabilities.

### 2.5 Coiled‐Coil XTEN Gels Promote Cell Survival Following In Vivo Transplantation

**Figure 6:**
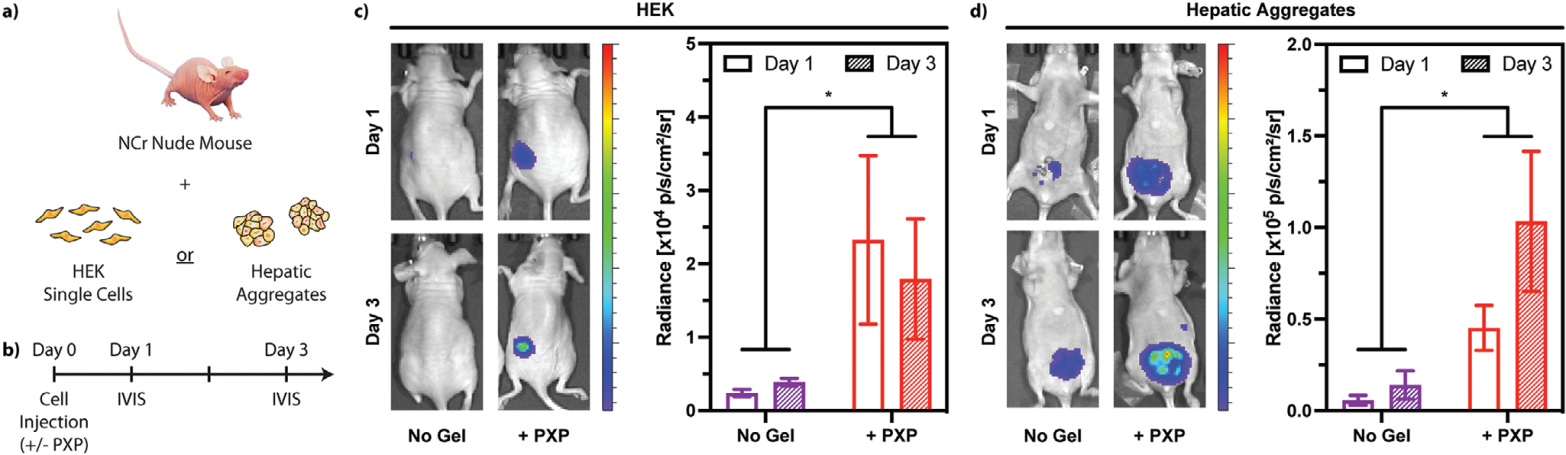
In vivo cell survival and function is enhanced when transplanted within injectable XTEN‐based coiled‐coil gels. a) Luciferase‐expressing HEK cells or hepatic aggregates were injected into Taconic NCr nude mice (4 per treatment group) with or without PXP gel and imaged via In Vivo Imaging System (IVIS) Spectrum imaging system 1 and 3 days post implantation. b) Timeline of the study. Results are shown for c) HEK cells constitutively expressing luciferase, and d) hepatic aggregates containing hepatocytes expressing luciferase downstream of a modified albumin promoter. Luminescence radiance was quantified and is reported as standard error about the mean (N = 4 biological replicates for each treatment group). An unpaired t‐test was implemented for injection condition comparison (* *p* < 0.05).

Encouraged by the coiled‐coil XTEN gels’ ability to protect cells upon syringe injection, we next sought to determine whether this supportive effect would translate into an in vivo setting. Here, we elected to transplant human 1) embryonic kidney (HEK) cells as a single‐cell suspension into the subcutaneous layer, and 2) hepatic aggregates comprising human hepatocytes and dermal fibroblasts into the perigonadal fat pad, each with or without PXP gel (5 wt%, 100 µL). Prior to transcutaneous transplantation through a syringe needle, cells were transduced to express firefly luciferase to enable cell survival and localization monitoring non‐invasively via In Vivo Imaging System (IVIS) Spectrum imaging; HEK cells constitutively expressed luciferase, whereas hepatocytes expressed luciferase downstream of a modified albumin promoter, for a surrogate measure of hepatic function. Cells were injected into NCr nude mice (Taconic, 4 per treatment group), an immunodeficient mouse model lacking mature T cells and known to tolerate human cell xenotransplantation. In both cell types, higher luminescence radiance was observed when cells were delivered within the PXP gel compared with those in DMEM media (**Figure** **6**), reflecting improved cell survival throughout syringe injection, more efficient engraftment, and/or enhanced cell proliferation in vivo when cells were encapsulated. Additionally, entrapment of the cells in the hydrogel mesh presents a physical barrier to systemic dissemination. This study showcases the tremendous promise that coiled‐coil XTEN gels hold in cell transplantation‐based therapies.

## 3 Conclusion

In this manuscript, we have introduced a telechelic recombinant protein‐based hydrogel based on XTEN. These materials are physically stabilized through non‐covalent coiled‐coil interactions, enabling both rapid shear‐thinning and self‐healing properties that suppose easy injection. Point mutations can be introduced within the coil domain that alternatively stabilize their homopentameric bundling, affording genetically encoded bulk hydrogels with user‐programmable viscoelasticity and rates of erosion. Demonstrating the protective nature of these engineered recombinant protein‐based biomaterials against membrane‐damaging extensional flow, we established the materials’ ability to protect and sustain viability of human fibroblasts, hepatic aggregates, HEK, and hESC‐CMs in culture following injection, both in vitro and in vivo. Looking forward, we anticipate that these injectable XTEN‐based hydrogels will provide great utility for both 3D cell culture and therapeutic cell transplantation.

## 4 Experimental Section

### Plasmid Construction

The original plasmid encoding for PXP (‐RGD) (a truncated version of XTEN with only 144 amino acid residues, flanked by identical P domains and 6xHis tags) was ordered and used as received from GenScript in a pQE‐30 backbone (T5 promoter, ampicillin and chloramphenicol acetyltransferase resistance). Cell adhesion sites (GRGDS sequences) were cloned into regions between the P domains and 6xHis tags on both ends resulting in PXP. Insertion at the N terminus was implemented through annealed oligo cloning with BamHI/SalI restriction sites (oligos from Integrated DNA Technologies, IDT). At the C terminus, Gibson Assembly of a GRGDS Gblock with overhangs was inserted at the HindIII/XhoI restriction sites (gBlock from IDT).

For the introduction of point mutations in P domains, plasmids encoding for P domains in a pTwist Amp High Copy cloning vector (ampicillin resistance) were purchased from Twist Biosciences and a plasmid encoding for X_144_ (XTEN with 144 residues) was ordered and used as received from GenScript in a pQE‐30 backbone. Point mutations (T40A, Q54A, or both T40A+Q54A) were introduced by site‐directed mutagenesis on P domain plasmids (primers from IDT). Mutated P domains were digested and inserted into the X_144_ plasmid at N terminal restriction sites BamHI/SalI and C terminal restriction sites XhoI/HindIII. All cloning was confirmed by Sanger Sequencing (Genewiz, Inc.) and final protein amino acid sequences can be found in Table S1, Supporting Information.

### Protein Expression, Purification, and Validation

Plasmids were transformed into BL21(DE3) *E. coli* cells and protein was expressed in autoinducing media [42.3 mm Na_2_HPO_4_, 22.04 mm KH_2_PO_4_, 0.28 m tryptone, 18.23 mm yeast extract, 85.56 mm NaCl, 2.78 mM glucose, 5.84 mm lactose, 0.6% (v/v) glycerol, pH 7.2] supplemented with 0.1 mg mL^−1^ carbenicillin at 37 °C for 6 – 8 h followed by 18 °C for 14 – 16 h with shaking rates of 200 rpm. Cell cultures were centrifuged, and cell pellets were collected and stored at −80 °C until purification.

Cell pellets were resuspended in equilibration buffer and lysed by sonication on ice (30% amplitude and 33% duty cycle, 1 s on 2 s off). Cell lysate was centrifuged, and the supernatant (clarified lysate) was then purified by Ni‐NTA affinity chromatography at room temperature. PXP and mutants (T40A, Q54A, and T40A+Q54A) were purified under standard conditions (equilibration buffer: 20 mm Tris, 50 mm NaCl, 10 mm imidazole, pH 8.0; wash buffer: 20 mm Tris, 50 mm NaCl, 15 mm imidazole, 0.1 pH 8.0; elution buffer: 20 mm Tris, 50 mm NaCl, 250 mm imidazole, pH 8.0). To remove endotoxins, the protein‐loaded resin was washed (5×) with 5 column volumes (CV) of 0.1% Triton X‐114‐supplemented wash buffer followed by with 5 CV of wash buffer (5×). Finally, the protein was eluted with 2 CV of elution buffer (4×). The purified protein was dialyzed against deionized water, sterile filtered, flash frozen with liquid nitrogen, and lyophilized to yield a white solid corresponding to the final product. Typical expression yields were exceptionally high, ≈100 mg of purified protein per L of bacterial culture.

Protein purity was assessed using sodium dodecyl sulphate‐polyacrylamide gel electrophoresis (SDS‐PAGE). Samples were diluted with 2X Laemmli sample buffer containing 2‐mercaptoethanol as a reducing agent and boiled at 100 °C for 10 min prior to loading on the gel. SDS‐PAGE was run in tris‐glycine running buffer at 130 V and stained with InVision His Tag stain (Thermo Fisher) followed by Coomassie stain. Using a QTRP 5600 Triple‐Quad time‐of‐flight mass spectrometer (AB SCIEX), we confirmed the molecular weight of each protein indicating successful expression and isolation of the protein of interest (Figures S1–S5, Supporting Information).

### Hydrogel Preparation

Following protein expression and purification, lyophilized PXP and coil mutant proteins were resuspended in phosphate‐buffered saline (PBS, pH 7.4) at 10% (w/v). Gels were vortexed, centrifuged, incubated at 37 °C for 10 min, and gently rocked at 4 °C overnight to encourage uniform gel formation. For cell encapsulation studies, lyophilized protein was rehydrated in cell suspension (10% w/v in DMEM) and incubated for 1 h at 37^ ^°C (or until gels were uniform), and gently mixed by stirring with a pipette tip.

### Rheological Characterization of Hydrogel Viscoelasticity

Characterization of material properties was performed using an Anton Paar Physica MCR 301 Rheometer with a parallel‐plate geometry (8 mm plate diameter, 500 µm gap) and a Peltier plate for temperature control. Once the geometry reached the measurement position, mineral oil was applied to the surrounding edges of the gel to prevent evaporation. Preformed gels of 30 µL were used in rheological analysis following a protocol adapted from the Burdick group.^[^ ^51^ ^]^ The first test included a 200 s oscillatory time sweep at constant strain (5%) and frequency (30 rad s^−1^) to ensure proper mixing of the gel and reach the plateau storage modulus. Next, an angular frequency sweep was performed at constant strain (5%) with varied frequency (0.1 – 100 rad s^−1^) to identify the linear LVER, followed by another time sweep to reset the gel. Then a strain sweep was implemented at constant frequency (30 rad s^−1^) with varied strain (0 – 500%), followed by another time sweep to reset the gel. Subsequently, a cyclic strain sweep test was employed by toggling between low (5% strain, 30 min, within LVER) and high (500% strain, 1 min, outside of LVER) strain four times at constant angular frequency (30 rad s^−1^, within LVER). Finally, a rotational shear thinning test at increasing shear rate (0.1 – 50 s^−1^) was implemented to demonstrate decreasing viscosity with increasing shear. Each gel type (PXP, T40A, Q54A, and T40A+Q54A) was analyzed in experimental quintuplicate. All tests were completed at both 25 °C (relevant for injection temperature) followed by 37^ ^°C (body temperature), each using a distinct set of gels.

Analysis of rheology data was automated in Python (code available on GitHub^[^ ^52^ ^]^) to calculate the average storage modulus, strain crossover, frequency crossover, and recovery time for each gel and condition. The storage modulus was calculated as the average of the last 25 data points in the first and second time sweeps. Strain and frequency crossovers were interpolated to determine when G″ (loss modulus) > G′ (storage modulus) during the corresponding strain and frequency sweep tests. Recovery time crossover was interpolated as the time it takes to recover back to the gel state (G′ > G″) after periods of high strain during the cyclic strain sweep test. A multiple comparisons two‐way ANOVA table was applied for statistical analysis to determine significance between mutant types.

### Gel Erosion

For physical erosion studies, 50 µL gels (*n* = 3 per gel type) at 10% (w/v) were formed in the bottom of 15 mL Falcon conical tubes and erosion was characterized in either PBS + 0.75 mm phenylmethylsulfonyl fluoride (PMSF) or Dulbecco's Modified Eagle Medium (DMEM) + 10% FBS + 1% Penicillin/Streptomycin. First, gels were washed twice with the relevant solution to remove any initially unincorporated protein. 10 mL of fresh solution was replaced on top of the gel and the tubes were incubated at 37 °C for the remainder of the study. Time points were taken every 24 h by centrifugation of samples at 200 × g for 1 min followed by imaging and removal of 100 µL supernatant for later analysis. To replace the fluid removed, 100 µL fresh solution was added back to each sample at each time point. The test continued for 12 days at which point some amount of intact gel remained visible at the bottom of each tube.

Protein concentration in the supernatant at each time point was used to determine extent of gel erosion in PBS + 0.75 mm PMSF. A bicinchoninic acid (BCA) assay with a standard curve of known protein concentrations of the corresponding coil proteins ranging from 5 – 700 µg mL^−1^ was utilized to quantify protein concentration in the samples. Samples were measured in technical duplicates on a 96‐well plate, and absorbance was detected (*λ*_abs_ = 562 nm) on a plate reader. Values were adjusted based on the total protein in each wash sample, the amount of protein removed at each time point, and evaporation in the tubes throughout the course of the study to obtain the final values for analysis.

### Cell Injection Protection and 3D Culture

NIH 3T3 Fibroblasts gifted from the Dr. Jennifer Davis lab (University of Washington) were thawed and suspended as 5 × 10^6^ cells per mL in media (DMEM‐no phenol red supplemented with 10% Fetal Bovine Serum and 1% Penicillin/Streptomycin). Human hESC‐CMs were thawed and suspended as 10 × 10^6^ cells per mL in media (RPMI‐no phenol red supplemented with 10% Fetal Bovine Serum, 2% B‐27, and 1% Penicillin/Streptomycin). Cell suspensions (Fibroblast or hESC‐CM) were added directly to lyophilized protein (PXP, T40A, Q54A, or T40A + Q54A) resulting in 10% (w/w) gels. Encapsulated cells were injected in triplicate at a volume of 20 µL per well (384‐well plate) through a 26G needle. Cells only (“no gel” condition) were injected in triplicate at a volume of 20 µL per well at a lower cell concentration of 1 × 10^5^ fibroblasts per mL or 1.25 × 10^5^ hESC‐CMs per mL. For comparison, cells that had not experienced encapsulation in gel or injection (“no injection” condition) were also plated at the same concentrations at the “no gel” condition. 20 µL of fresh media was added to each well, then the plate was incubated for 24 h at 37 °C. Cells were directly stained in media resulting in a final concentration of 4 µm CalceinAM (*λ*_excitation_ = 488 nm, *λ*_emission_ = 520 nm) and 8 µm Ethidium Homodimer‐1 (*λ*_excitation_ = 580 nm, *λ*_emission_ = 604 nm) then imaged on a Leica Stellaris 5 Confocal through the full thickness of each sample with a step size of 3 um in the z direction. Viability was quantified using the Leica Application Suite X (LAS X) software by implementation of an Otsu threshold for both channels (CalceinAM and Ethidium Homodimer‐1). Cells were incubated at 37 °C for an additional 48 h, and final cell viability of each well was analyzed using an NC200TM NucleoCounter. A multiple comparisons one‐way ANOVA table was implemented for statistical analysis.

### Generation of Firefly Luciferase‐Expressing HEK Cells

Human embryonic kidney (HEK293T) cells were plated at ≈40% confluency and allowed to adhere to tissue culture plastic overnight. Fresh media was added, and then HEKs were transfected with envelope plasmid pMD2.G (Addgene #12 259), packaging plasmids pMDLG/pRRE (Addgene #12 251/) and pRSV‐REV (Addgene #12 253), and transfer plasmid pLX307 (Addgene #117 734) containing Elongation factor 1‐alpha 1 (EF1a1)‐driven expression Firefly Luciferase, using Lipofectamine 2000 (Invitrogen). Cells were cultured for 2 days post‐transfection, and virus‐laden media was harvested. Viral media was filtered (0.45 µm) and tested using lentiviral titration card (ABM Biologics). Active lentivirus was concentrated by mixing viral media with 4X lentiviral concentration solution (40% w/v PEG‐8000, 1.2 m NaCl), vigorously shaking for 60 s, and agitated overnight at 4 °C. The following day, flocculated lentiviral particles were pelleted at 1600×g for 60 min at 4 °C, and supernatant was aspirated. Pellet was resuspended at 10× relative to initial viral media volume in PBS.

Fresh HEK cells were plated at ≈40% confluency and allowed to adhere to tissue culture plastic overnight. Media was replaced the following day and concentrated virus was added. HEKs were incubated overnight with lentiviral particles, and supernatant media was aspirated the following day. Fresh media was added and cells were allowed to recover for an additional 24 h. HEKs were then passaged such that their final confluency was ≈50% and allowed to adhere overnight. Cells were selected using 6 µg mL^−1^ of Puromycin (Invitrogen) for 24 h. Puromycin‐laden media was removed and from this point HEKs were cultured following standard procedures.

### Hepatocyte Transduction and Aggregation

Cryopreserved human hepatocytes (Gibco) were thawed and then immediately transduced in suspension culture with a lentiviral vector expressing firefly luciferase under the albumin promoter (pTRIP.Alb.IVSb.IRES.tagRFP‐DEST, provided through a Materials Transfer Agreement with Charles Rice, The Rockefeller University). The concentrated virus was diluted 1:5 in hepatocyte medium containing N‐2‐hydroxyethylpiperazine‐N‐2‐ethane sulfonic acid buffer (HEPES; 20 mM; Gibco) and 4 µm mL^−1^ polybrene (AmericanBio), and incubated with human hepatocytes in ultra‐low attachment 6‐well plates (Corning) for 6 h. After incubation, the transduced hepatocytes were collected for aggregation along with normal human dermal fibroblasts (NHDFs) at a 1:1.6 ratio following previously reported procedures.^[^ ^53^ ^]^ To induce aggregation, hepatocytes and fibroblasts were plated in AggreWell Micromolds (400 µm square AggreWell micromolds, Stem Cell Technologies) in high‐glucose DMEM (Corning) containing 10% (v/v) FBS (Gibco), 1% (v/v) insulin, transferrin, sodium selenite supplement (BD Biosciences), 7 ng mL^−1^ glucagon (Sigma), 0.04 ug mL^−1^ dexamethasone (Sigma), and 1% (v/v) pen‐strep (Invitrogen) and cultured overnight.

### In Vivo Implantation in Mice and IVIS Imaging

HEK cells were cultured to confluency and harvested in single‐cell suspension in unsupplemented DMEM the morning of surgery. The HEK cell suspension was added to lyophilized PXP protein to give 1 × 10^7^ HEKs per mL of 5 wt% gel, and the mixture was left at 37 °C for ≈1.5 h to allow for gel formation. The mixture was gently stirred with a pipette tip intermittently to promote homogenous gel formation and cell dispersion. The remaining HEK cell suspension was kept on ice during his time. HEK cell suspension at 1 × 10^7^ cells mL^−1^ and HEK cells + PXP were backloaded into a syringe fitted with a 20‐gauge (20G) needle.

Hepatic aggregates suspended in unsupplemented DMEM were added to lyophilized PXP protein to give ≈1 × 10^7^ hepatocytes and 1.6 × 10^7^ fibroblasts per mL of 5 wt% gel. The mixture was left on ice for ≈2 h to allow for gel formation with intermittent stirring as noted above. The remaining hepatic cluster suspension was kept on ice during this time, then both mixtures were loaded into a syringe fitted with a 20G needle.

All surgical procedures were approved by the University of Washington Animal Care and Use Committee. Taconic NCr nude mice (female, 9–10 weeks old, four mice per treatment group) were anesthetized using isofluorane. 100 µL cell‐laden gels or cell suspension in DMEM basal media was injected into the subcutaneous space through a 20G needle (HEKs) or perigonadal fat pad via a small incision (hepatic aggregates). The incision was closed aseptically, and animal recovery from surgery was monitored. Animals were administered meloxicam (5 mg kg^−1^) subcutaneously post operation and again at 1 and 2 days following surgery. After 1‐ and 3‐days post‐operation, mice were administrated D‐luciferin solution (250 µL, 15 mg mL^−1^) and imaged using In Vivo Imaging System (IVIS) Spectrum imaging system (PerkinElmer) per manufacturer's protocol.

## Conflict of Interest

Some of this work was done while Charles E. Murry was an employee of and equity holder in Sana Biotechnology. This work does not overlap with Sana's scientific activities. The remaining authors declare no competing financial interests.

## Author Contributions

J.I.B. and M.O.B. contributed equally to this work. For this manuscript, J.I.B., M.O.B., N.E.G., P.B.R., and C.A.D. conceived and designed the experiments; J.I.B., M.O.B., and N.E.G. performed experiments related to protein expression, materials mechanical characterization, and erosion studies. R.D.K. performed circular dichroism spectroscopy and analysis. J.W.H. created the luciferase‐expressing HEK cells. K.R.S., F.Z., O.P., and N.E.G. performed the in vivo studies and analysis. J.I.B., M.O.B., N.E.G., F.Z., R.D.K., and C.A.D. analyzed the data and prepared the figures; J.I.B. and C.A.D. spearheaded the paper writing efforts with feedback from all other authors; C.E.M., K.R.S., and C.A.D. provided mentorship, lab space, and funding.

## Supporting information

Supporting Information

## Data Availability

The data that support the findings of this study are available in the supplementary material of this article.
