# Supplementary material for: Genetically Encoded XTEN‐based Hydrogels with Tunable Viscoelasticity and Biodegradability for Injectable Cell Therapies
Source: Adv Sci (Weinh). 2024 Mar 13;11(24):2301708. doi: 10.1002/advs.202301708 (PMC11200090; doi:10.1002/advs.202301708)
Supplement: Supplementary file 1 — Supporting Information [file ADVS-11-2301708-s001.pdf]

ADVANCED SCIENCE
Open Access

# Supporting Information

for *Adv. Sci.*, DOI 10.1002/advs.202301708

Genetically Encoded XTEN-based Hydrogels with Tunable Viscoelasticity and Biodegradability for Injectable Cell Therapies

*Jennifer I. Bennett, Mary O'Kelly Boit, Nicole E. Gregorio, Fan Zhang, Ryan D. Kibler, Jack W. Hoye, Olivia Prado, Peter B. Rapp, Charles E. Murry, Kelly R. Stevens and Cole A. DeForest**

Supplementary Information for

# Genetically Encoded XTEN-based Hydrogels with Tunable Viscoelasticity and Biodegradability for Injectable Cell Therapies

Jennifer I. Bennett, Mary O'Kelly Boit, Nicole E. Gregorio, Fan Zhang, Ryan D. Kibler, Jack W. Hoye, Peter B. Rapp, Charles E. Murry, Kelly R. Stevens, Cole A. DeForest*

*Correspondence to: profcole@uw.edu

**Table of Contents**


General synthetic information .......... 3

Table S1 Amino acid sequences for proteins used in this work .......... 4

Figure S1 PXP (-RGD) verification .......... 5

Figure S2 PXP verification .......... 6

Figure S3 T40A verification .......... 7

Figure S4 Q54A verification .......... 8

Figure S5 T40A+Q54A verification .......... 9

Figure S6 Circular dichroism (CD) measurements of PXP, T40A, Q54A, and T40A+Q54A .. 10

Figure S7 Strain and frequency sweeps at 25 °C and shear thinning tests at 25 °C and 37 °C 11

Figure S8 Physical Erosion of XTEN-based coiled-coil gels in cell culture medium .......... 12

References .......... 13

## General synthetic information

Chemical reagents and solvents were purchased from either Sigma-Aldrich or Fisher Scientific and used as received unless otherwise noted. Deionized water ($dH_2O$) was generated by a U.S. Filter Corporation Reverse Osmosis System with a Desal membrane. Lyophilization was performed on a LABCONCO FreeZone 2.5 Plus freeze-dryer equipped with a LABCONCO rotary vane 117 vacuum pump. Matrix-assisted laser desorption/ionization time of flight (MALDI-TOF) mass spectrometry was performed in reflectron positive ion mode or reflectron negative ion mode on a Bruker AutoFlex II using a matrix of α-cyano-4-hydroxycinnamic acid:2,5-dihydroxy benzoic acid (2:1). Whole-protein mass spectrometry was performed using a Waters Synapt – G2 QTOF. Confocal microscopy was performed on a Leica Stellaris 5. Polymerase chain reaction (PCR) was performed in a Bioer LifeECO thermal cycler. Protein expression was performed in a Thermo Scientific MaxQ 4000 shaker incubator. Cells were lysed using a Fisher Scientific Model 505 Sonic Dismembrator with a 1.27 cm diameter probe. UV-Vis assays were performed on a BioTek Synergy H1M plate reader. Fluorescent and true color gel imaging was performed on an Azure 600 AZI600 scanner. Rheological measurements were performed on an Anton Paar Physica MCR301 equipped with a C-PTD200 Peltier plate and a parallel plate geometry (8 mm diameter). Mammalian cell culture was performed in a NuAire LabGard ES NU-437 Class II Type A2 Biosafety Cabinet. Cells were maintained in a Sanyo inCu saFe® MCO-17AC incubator at 37 °C and 5% $CO_2$.

**Table S1 Amino acid sequences for proteins used in this work**

Below is a list of protein amino acid sequences used in this work. XTEN is highlighted in orange, P domains are highlighted in blue, point mutations are highlighted and underlined in green, and RGD cell adhesion sites are highlighted in red.

PXP:

MRGSHHHHHHGSGRGDSVDGSGSGSGSGSGSGAPQMLRELQETNAALQDVRELLRQQVKEITFLKNTVMESDASGSG
SGSGSGSGSLDGSPAGSPTSTEEGTSESATPESGPGTSTEPSEGSAPGSPAGSPTSTEEGTSTEPSEGSAPGTSTEP
SEGSAPGTSESATPESGPGSEPATSGSETPGSEPATSGSETPGSPAGSPTSTEEGTSESATPESGPGTSTEPSEGSA
PLDGSGSGSGSGSGSGAPQMLRELQETNAALQDVRELLRQQVKEITFLKNTVMESDASGSGSGSGSGSGSLEGRGDS
HHHHHHKLN

T40A:

MRGSHHHHHHGSGRGDSGSGSGSGSGSGSGAPQMLRELQEANAALQDVRELLRQQVKEITFLKNTVMESDASGSGSG
SGSGSVDGSPAGSPTSTEEGTSESATPESGPGTSTEPSEGSAPGSPAGSPTSTEEGTSTEPSEGSAPGTSTEPSEGS
APGTSESATPESGPGSEPATSGSETPGSEPATSGSETPGSPAGSPTSTEEGTSESATPESGPGTSTEPSEGSAPLES
GSGSGSGSGSGAPQMLRELQEANAALQDVRELLRQQVKEITFLKNTVMESDASGSGSGSGSGSGSGRGDSHHHHHHK
LN

Q54A:

MRGSHHHHHHGSGRGDSGSGSGSGSGSGSGAPQMLRELQETNAALQDVRELLRQAVKEITFLKNTVMESDASGSGSG
SGSGSVDGSPAGSPTSTEEGTSESATPESGPGTSTEPSEGSAPGSPAGSPTSTEEGTSTEPSEGSAPGTSTEPSEGS
APGTSESATPESGPGSEPATSGSETPGSEPATSGSETPGSPAGSPTSTEEGTSESATPESGPGTSTEPSEGSAPLES
GSGSGSGSGSGAPQMLRELQETNAALQDVRELLRQAVKEITFLKNTVMESDASGSGSGSGSGSGSGRGDSHHHHHHK
LN

T40A+Q54A:

MRGSHHHHHHGSGRGDSGSGSGSGSGSGSGAPQMLRELQEANAALQDVRELLRQAVKEITFLKNTVMESDASGSGSG
SGSGSVDGSPAGSPTSTEEGTSESATPESGPGTSTEPSEGSAPGSPAGSPTSTEEGTSTEPSEGSAPGTSTEPSEGS
APGTSESATPESGPGSEPATSGSETPGSEPATSGSETPGSPAGSPTSTEEGTSESATPESGPGTSTEPSEGSAPLES
GSGSGSGSGSGAPQMLRELQEANAALQDVRELLRQAVKEITFLKNTVMESDASGSGSGSGSGSGSGRGDSHHHHHHK
LN

**Figure S1 PXP (-RGD) verification**

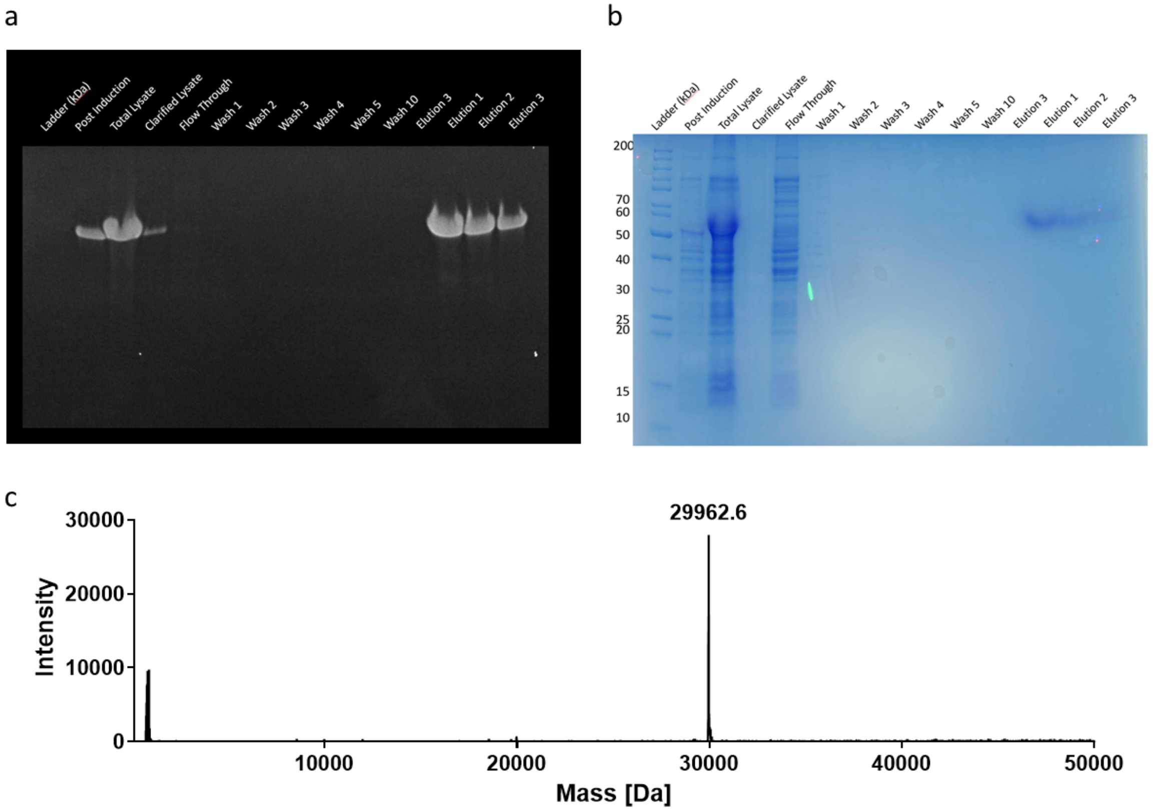


SDS-PAGE analysis indicated successful isolation of a single product from Ni-NTA purification, as evidenced by a single band in the elution lanes corresponding to an apparent molecular weight of 60 kDa. The final product ran high, as expected from literature precedent, due to weak binding to SDS caused by a lack of hydrophobic amino acids in XTEN[1]. a) InVision$^{TM}$ His-tag-stained SDS-PAGE. b) Coomassie-stained SDS-PAGE. c) Mass spectrometry reported a final mass of 29,962.6 Da (calculated mass is 29,948.2 Da).

**Figure S2 PXP verification**

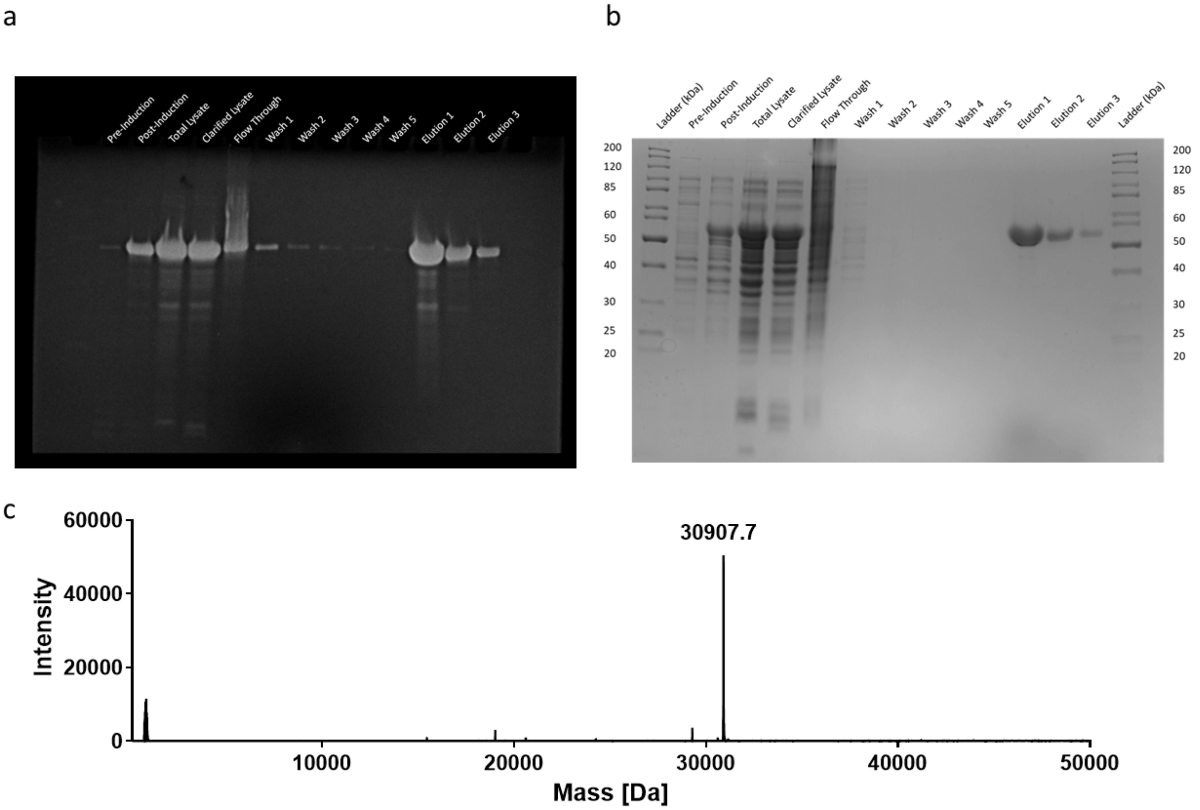


SDS-PAGE analysis indicated successful isolation of a single product from Ni-NTA purification, as evidenced by a single band in the elution lanes corresponding to an apparent molecular weight of 60 kDa. The final product ran high, as expected from literature precedent, due to weak binding to SDS caused by a lack of hydrophobic amino acids in XTEN[1]. a) InVision$^{TM}$ His-tag-stained SDS-PAGE. b) Coomassie-stained SDS-PAGE. c) Mass spectrometry reported a final mass of 30,907.7 Da (calculated mass is 30,893.8 Da).

**Figure S3 T40A verification**

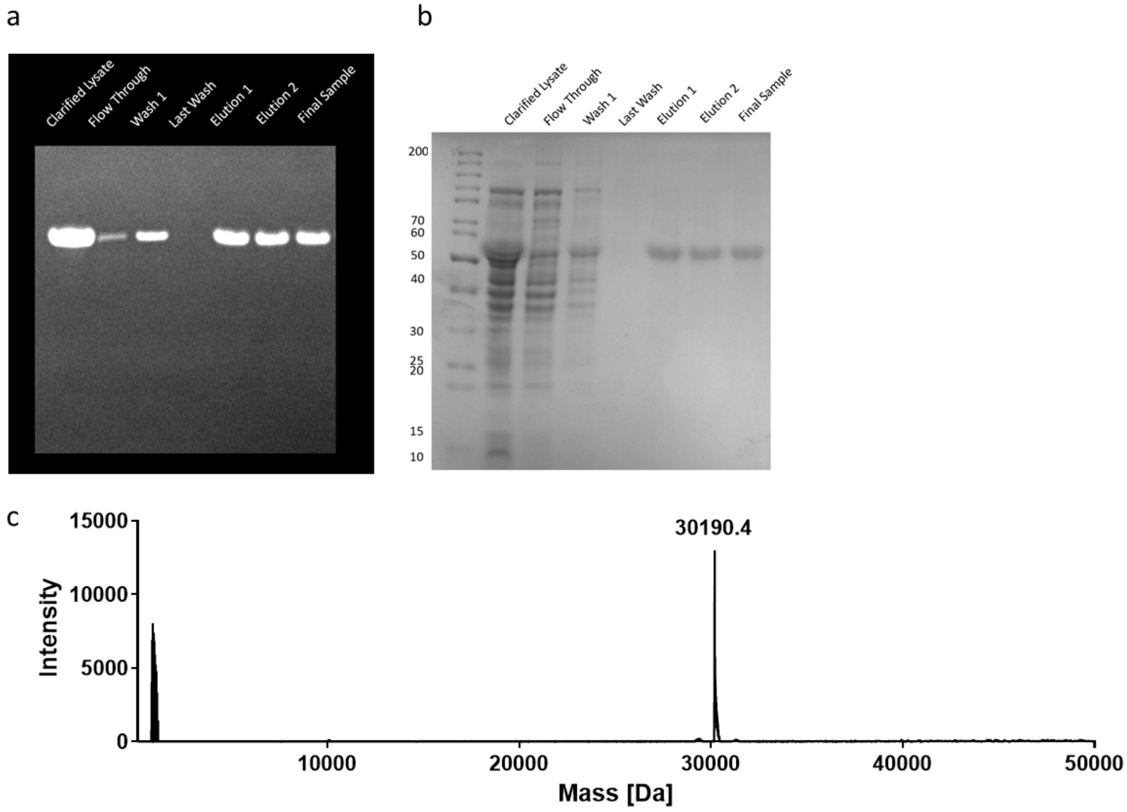


SDS-PAGE analysis indicated successful isolation of a single product from Ni-NTA purification, as evidenced by a single band in the elution lanes corresponding to an apparent molecular weight of 60 kDa. The final product ran high, as expected from literature precedent, due to weak binding to SDS caused by a lack of hydrophobic amino acids in XTEN[1]. a) InVision$^{TM}$ His-tag-stained SDS-PAGE. b) Coomassie-stained SDS-PAGE. c) Mass spectrometry reported a final mass of 30,190.4 Da (calculated mass is 30,176.1 Da).

**Figure S4 Q54A verification**

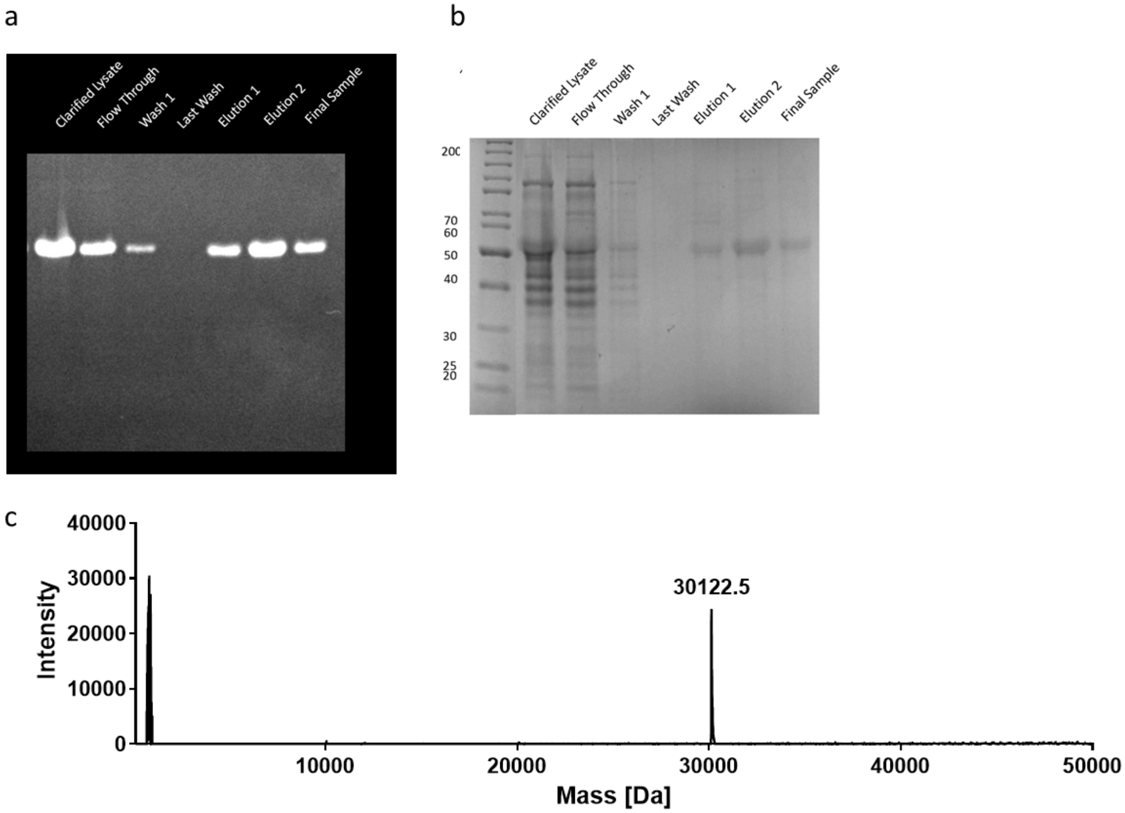


SDS-PAGE analysis indicated successful isolation of a single product from Ni-NTA purification, as evidenced by a single band in the elution lanes corresponding to an apparent molecular weight of 60 kDa. The final product ran high, as expected from literature precedent, due to weak binding to SDS caused by a lack of hydrophobic amino acids in XTEN[1]. a) InVision$^{TM}$ His-tag-stained SDS-PAGE. b) Coomassie-stained SDS-PAGE. c) Mass spectrometry reported a final mass of 30,122.5 Da (calculated mass is 30,122.1 Da).

**Figure S5 T40A+Q54A verification**

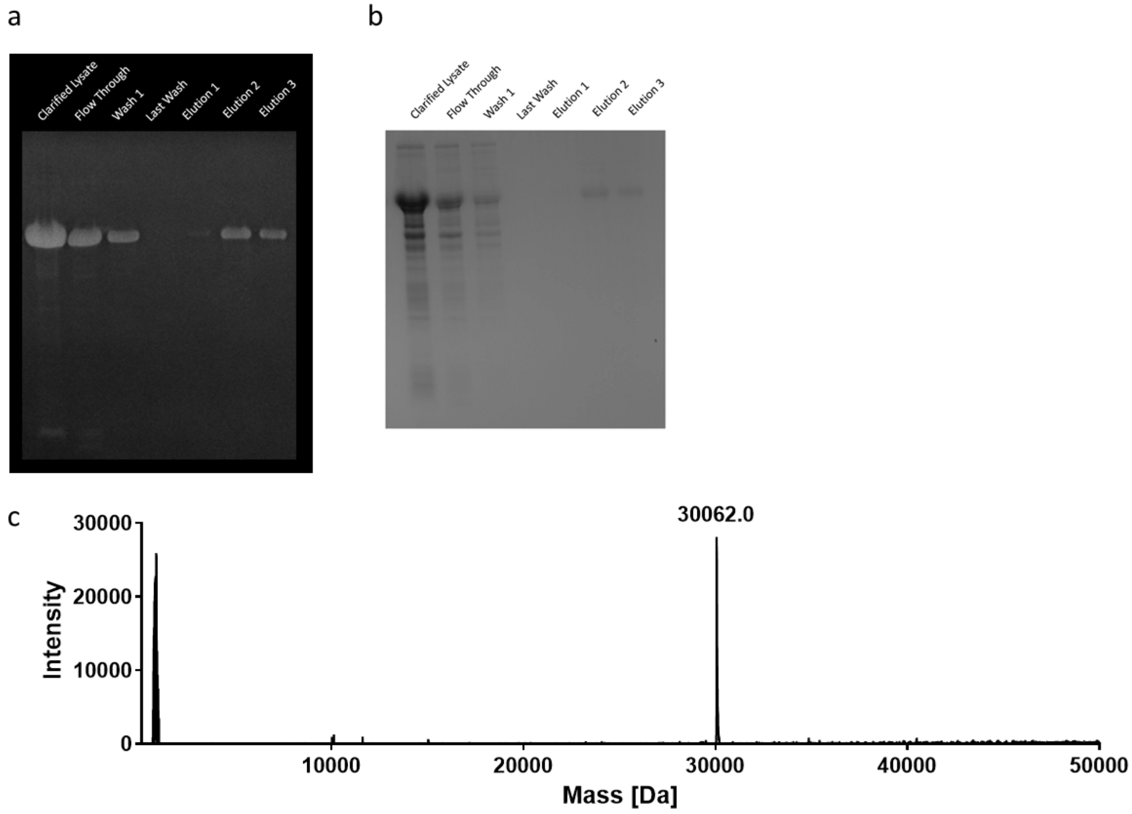


SDS-PAGE analysis indicated successful isolation of a single product Ni-NTA purification, as evidenced by a single band in the elution lanes corresponding to an apparent molecular weight of 60 kDa. The final product ran high due to weak binding to SDS caused by a lack of hydrophobic amino acids in XTEN[1]. a) InVision™ His-tag-stained SDS-PAGE. b) Coomassie-stained SDS-PAGE. c) Mass spectrometry reported a final mass of 30,062.0 Da (calculated mass is 30,062.0 Da).

**Figure S6 Circular dichroism (CD) measurements of PXP, T40A, Q54A, and T40A+Q54A**

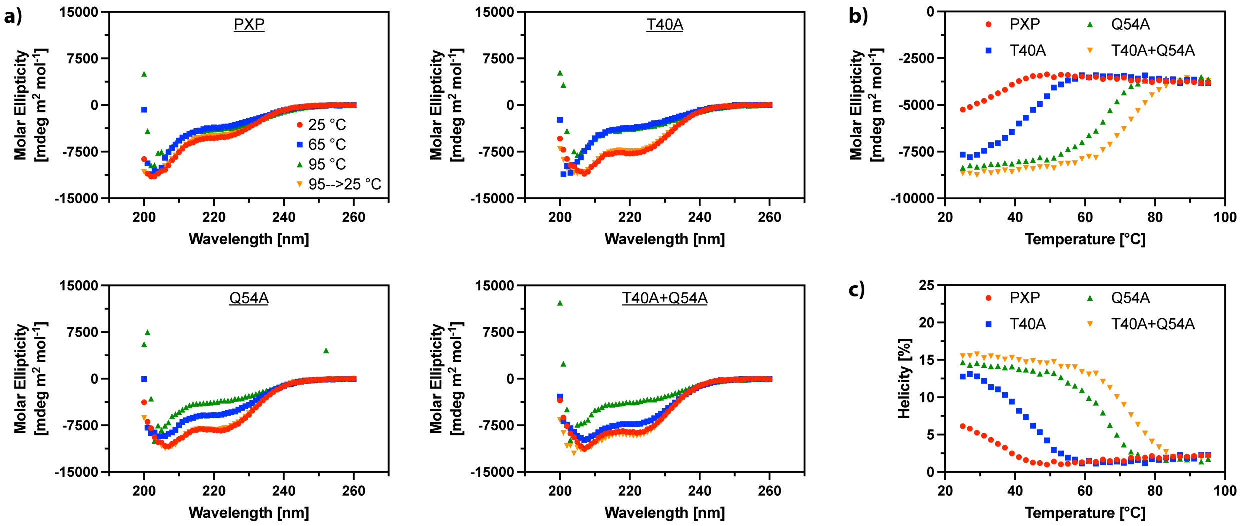


Circular dichroism (CD) measurements were performed using an JASCO model J-1500 CD spectrometer with a multi-cell holder on protein samples diluted to 0.4 mg $mL^{-1}$ in PBS pH 7.4 in 0.1 cm cuvettes. Temperature melts (25 – 95 °C) were carried out at a heating rate of 2.5 °C/min and monitored by the change in ellipticity at 222 nm at a 2 °C interval. Wavelength scans (260 - 190 nm) were performed during temperature melts at 25 °C, 65 °C, and 95 °C. Temperature was held constant during wavelength scans. The scanning increment was 1 nm at 100 nm $min^{-1}$ and an integration time of 2 s. A final scan was performed after cooling the sample back to 25 °C. Percent helicity was determined by normalizing the molar ellipticity at 222 nm to between -3000 (unfolded) and -39500 (folded) mdeg x $m^2$ $mol^{-1}$.

(a) CD wavelength scans for the four constructs at 25 (red circle), 65 (blue square), and 95 °C (green triangle) taken during thermal melts. A final scan at 25 °C after cooling (95-->25 °C, orange inverted triangle) represents a return to the same secondary structure content as was present before heating. (b) CD thermal melt data collected at 222 nm to track helical character between 25 and 95 °C. PXP in red circle, T40A in blue square, Q54A in green triangle, T40A+Q54A in orange inverted triangle. (c) Transformation of the data in (b) to quantify the helical character (vs random coil). 15% helicity at 25 °C agrees with PSIPRED analysis[2] of the T40A+Q54A sequence.

**Figure S7 Strain and frequency sweeps at 25 °C and shear thinning tests at 25 °C and 37 °C**

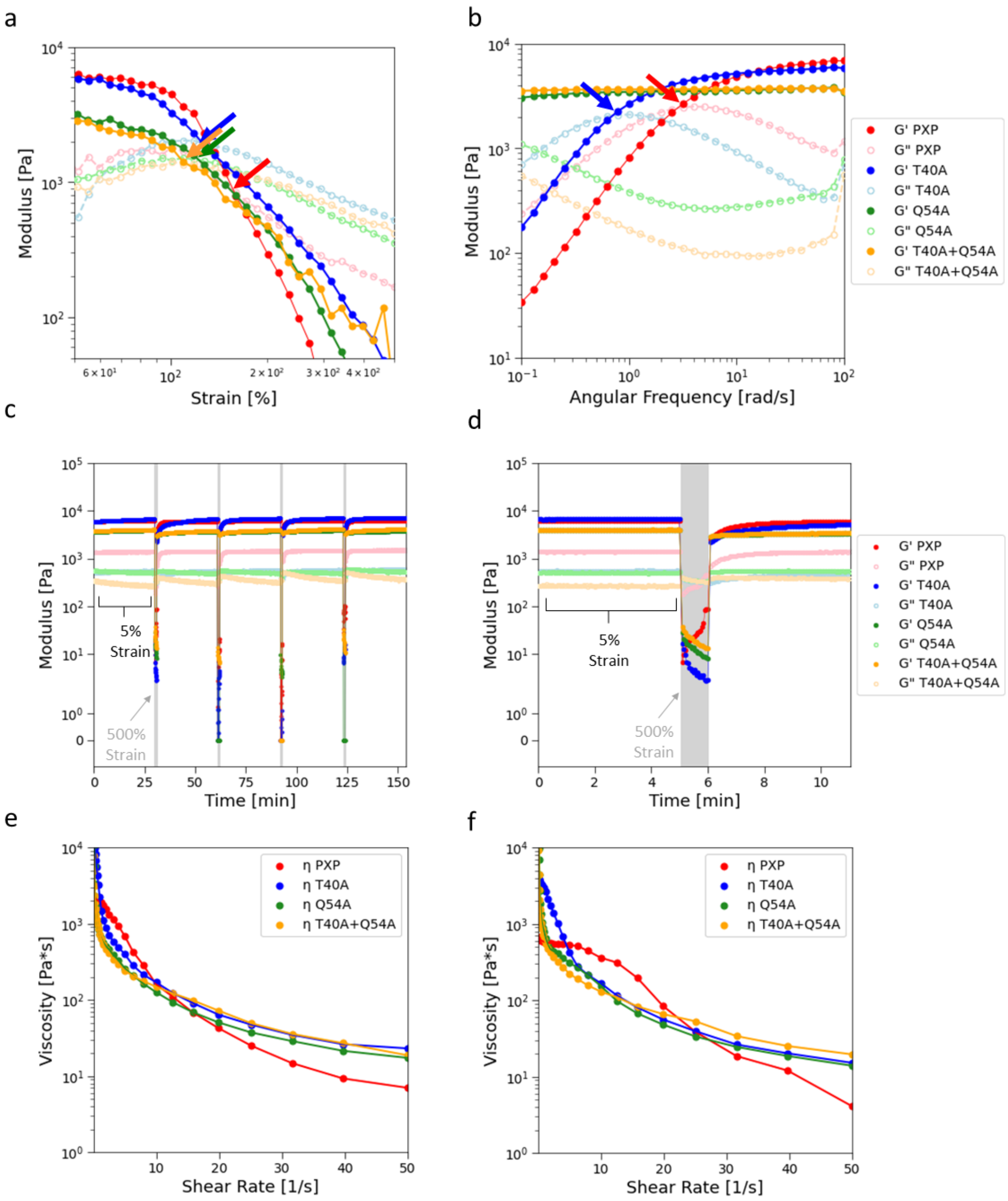


Strain and frequency sweeps at 25 °C and shear thinning tests at 25 °C and 37 °C. PXP in red, T40A in blue, Q54A in green, and T40A + Q54A in orange. a-d) G': storage modulus represented by dark-colored closed circles, G": loss modulus represented by light-colored open circles. a) Representative strain sweep at 25 °C (30 rad $s^{-1}$, 0 – 500% strain). b) Representative frequency sweep at 25 °C (5% strain, 0.1 – 100 rad $s^{-1}$). c) Representative cyclic strain sweep at 25 °C (30 rad $s^{-1}$, 5% low strain, 500% high strain in gray) demonstrating full recovery of all gels after 4 periods of high strain. d) Zoom on a representative high strain cycle of the cyclic strain sweep. e) Representative rotational shear thinning test at room temperature (25 °C) indicating lowered viscosity at increased shear rates. f) Representative rotational shear thinning test at physiological temperature (37 °C).

**Figure S8 Physical Erosion of XTEN-based coiled-coil gels in cell culture medium**

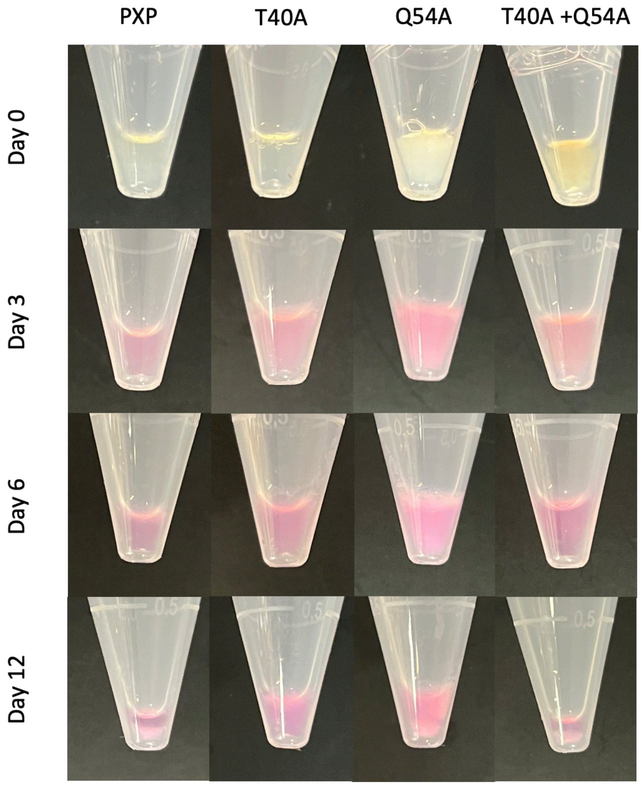


Physical erosion of XTEN-based coiled-coil gels in cell culture medium. Photographs of each gel type following 0, 3, 6, and 12 days in Dulbecco's Modified Eagle Medium (DMEM) at 37 °C.

## References

[1] V. N. Podust, S. Balan, B. C. Sim, M. P. Coyle, U. Ernst, R. T. Peters, V. Schellenberger, *Journal of Controlled Release* **2016**, *240*, 52.
[2] D. W. A. Buchan, D. T. Jones, *Nucleic Acids Research* **2019**, *47*, W402.
